# Modified Stoppa as an alternative surgical approach for fixation of anterior fracture acetabulum: a randomized control clinical trial

**DOI:** 10.1186/s13018-020-01660-3

**Published:** 2020-04-17

**Authors:** Ahmed Saleh Al Adawy, Abdel Hamid Abdel Aziz, Faisal Ahmed El Sherief, Wael Shaban Mahmoud, Mahmoud Mabrook, Yaser El-Sayed Hassan

**Affiliations:** 1 Department of Orthopedics, Nasr City Insurance Hospital, General Organization of Health Insurance, Cairo, Egypt; 2 Department of Orthopedics, Faculty of Medicine, Al-Azhar University, Cairo, Egypt

**Keywords:** Acetabular fracture, Approach, Complications, Reduction, Clinical outcome

## Abstract

**Background:**

Fracture acetabulum is a challenging, difficult to treat orthopedic injury due to its location and associated concomitant injuries. The modified Stoppa approach for reduction of fracture acetabulum improves access to quadrilateral surface and posterior column and is considered to be advantageous in many facets of the surgery.

**Methods:**

A randomized controlled clinical study was conducted to provide an update on our experience with modified Stoppa as a favorable surgical approach in acetabular fractures. In the period between 2015 and 2017; 18 patients with acetabular fractures operated by the classical ilioinguinal approach were retrospectively reviewed through their medical records as a controlled group and selected 20 patients with acetabular fractures were operated in the period between 2017 and 2019 using the modified Stoppa approach, as a clinical case group. The two groups were compared regarding operative data and postoperative clinical data, complications, and follow up. Cases were operated in Al Zahraa University Hospital and Nasr City Insurance Hospital by the same surgeon and one of the co-authors.

**Results:**

(Group A) those operated by ilioinguinal approach and (Group B) those operated using Stoppa approach. The whole study included 25 males (66%); mean age was 41.8 ± 8.42 (range 18-65) years. The mean follow-up period was 18.5 months with 5 patients lost to follow-up. Both column fractures were observed in most of the patients (45%). We observed anatomical reduction, excellent clinical outcome scores in 75% of patients of group B (*p* = 0.030), and less complications.

**Conclusion:**

Our findings indicated that the modified Stoppa approach is the most convenient approach when surgery is required and achieved favorable results in the treatment of anterior acetabular fractures because it improves visualization in lateral compression injuries and allows treatment of both column fractures with single incision. Hence, it is recommended as an alternative to ilioinguinal approach in developing countries. Further, larger-scale comparative studies of the two surgical modalities for different acetabular fracture types and long-term complications are recommended.

**Trial registration:**

A retrospective registration is proceeding through Clinicaltrials.gov.

**Level of evidence:**

Level III, therapeutic clinical study

## Introduction

Acetabular fracture is a serious orthopedic injury that is managed using anterior, posterior, extensile, and combined surgical approaches. The correct surgical approach is crucial for accurate reduction. Ilioinguinal approach and the modified Stoppa approach are commonly used anterior approaches. Initially, Rives et al. and Stoppa et al. employed the modified Stoppa approach in inguinal hernia surgery. Cole and Bolhofner and Hirvensalo et al. described it as a method to approach the anterior acetabulum and pelvic bone [1–4].

Modified Stoppa approach has been evaluated in many studies that reported similar clinical outcomes as the ilioinguinal approach [5–10]. It is advantageous in treating acetabular fractures with anterior column involvement and even considered a superior alternative to ilioinguinal approach [1–3]. The modified Stoppa approach has recently become popular in Europe as it is less invasive and provides better visualization to quadrilateral plate and the posterior column [1–3, 5]. It allows direct (medial) buttressing of fractures with associated central protrusion of the femoral head.

There is extensive evidence available on early radiographic results, but long-term follow-up data is not sufficient [2, 5–7]. In this study, we evaluated operative technique of Stoppa approach, its clinical outcomes and complications compared to the classical ilioinguinal approach with respect to comminution of fractures.

## Materials and methods

### Preoperative component

#### Patient selection

Inclusion criteria:

- Age of 18 to 65 years
- Anterior column acetabular fracture
- Anterior column with posterior hemitransverse (both columns)
- T type
- Within 4 weeks of trauma
- Willingness to participate in a strict follow-up and rehabilitation protocol

Exclusion criteria:

- Treatment with conservative skeletal traction
- Isolated posterior wall fractures
- Active infection
- Severe medical problem illegible for anesthesia
- Pathological fractures
- Open fractures of acetabulum
- Patients with severe osteoarthritis hip joint
- Non ambulatory patient
- Neglected fractures (more than 4 weeks).
- Patients unfit for surgery or refusing surgery
- Patients refused surgery

#### Preoperative precautions and preparation

- Informed consent: This was obtained from all patients, and the details of the surgical procedure including benefits, possible risks, complications, and follow-up protocol were clearly explained to the patients.
- Routine pre-operative laboratory investigations: Blood picture, blood sugar, bleeding profile, renal and liver functions for all patients.
- Electrocardiogram for all patients above 40 years of age.
- Reservation of 2 units of whole blood.
- Pre-operative antibiotics: All patients received one dose of 3rd generation (Cephalosporin has to be given within 1 h before incision).
- DVT (deep venous thrombosis) prophylaxis with Enoxaparin 40 mg SC/24 h, to be stopped 12 h before surgery.

**Fig. 1:**
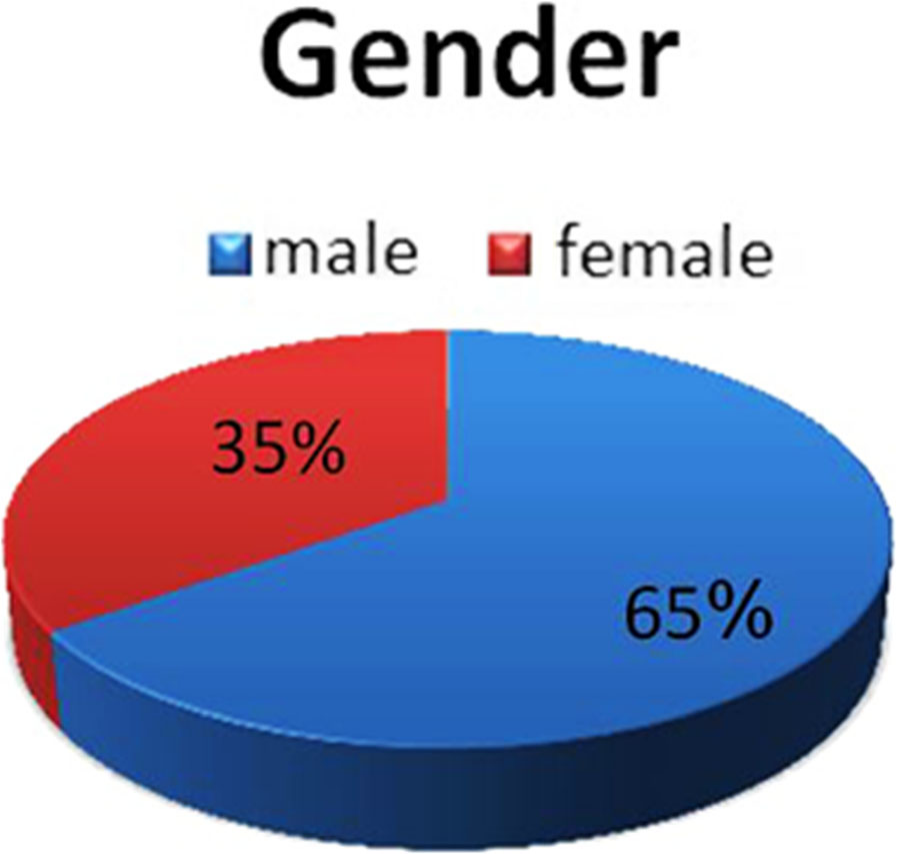
Gender distribution

A total of 88 patients with acetabular fractures attended trauma section at Al Zahraa University Hospital and Nasr City Insurance Hospital in Cairo since June 2015 until end of May 2019; 30 of them whose operated using ilioinguinal approach in the period of June 2015 until July 2017 and fit inclusion criteria underwent retrospective review of their medical records by one of the co-authors considering technique, time of operation, and blood loss. Postoperative follow up of complications was assessed through contacting those patients but only 18 patients could be reached were included in our study as a GROUP A (12 males, 6 females). While twelve patients excluded from the study as ineligible and 5 with pathological fracture, 3 patients refused operation, 7 unfit for anesthesia due to cardiac problems, 6 died from internal hemorrhage, and 5 refused to sign consent. Only 20 patients included as a GROUP B, 13 males (65%) and 7 females (35%) (Fig. 1), with acetabular fractures between 2017 and 2019 were considered in the study. Their mean age was 36.8 ± 8.42 (20-73) years (Table 1). The patients underwent open reduction and internal fixation by reconstruction plate using modified Stoppa approach operated by the same co-author at Al Zahraa University Hospital and Nasr City Insurance Hospital in Cairo. The mean follow-up period was 18.5 months with 5 patients lost to follow-up. In most of the patients, both column fractures (45%) were observed. The two groups: (Group A) 18 patients with acetabular fracture treated with ilioinguinal approach as per reviewed medical records and (Group B) 20 patients with modified Stoppa approach with their operative data. The American Society of Anesthesiologists (ASA) physical status classification (ASAPS) for both the groups and operative variables are illustrated in Tables 2 and 3.

**Table 1:** Demographic distribution between the two groups

Age group	(Group A)^*^ number	Percentage (%)	(Group B)*	Percentage (%)
18-30 years	3	16.6	5	25
31-60 years	11	61.1	14	70
> 60 years	4	22.2	1	5
Total	18	100	20	100
Mean ± SD	43 ± 5.23		36.8 ± 8.42	**
Median	33-67 years		18-65	
Male gender	12	67	13	65
Female gender	6	33	7	35

*Group A indicates 18 patients operated by ilioinguinal approach

*Group B indicates 20 patients operated via modified Stoppa approach

**p = 0.789 for age distribution, **p = 0.999 for gender distribution

**Table 2:** Collected patient data and the American Society of Anesthesiologists (ASA) physical status classification system (ASAPS)

	No. of cases	Age (year)	Gender	Comminution	ASAPS	Fracture type	Fixation method	Operative approach
Collected patient data (group B) clinical cases								
	1	26	Male	-	ASA 1	BC	P + S	M-S
	2	46	Male	-	ASA 2	TS	P + S	M-S
	3	26	Female	-	ASA 1	AC	P + S	M-S
	4	47	Male	C	ASA 2	BC	P + S	M-S + LW
	5	25	Female	-	ASA 1	BC	P + S	M-S
	6	31	Male	C	ASA 1	BC	P + S + W	M-S + LW
	7	42	Male	-	ASA 2	AC	P + S	M-S
	8	40	Male	C	ASA 1	TS	P + W	M-S + LW
	9	69	Male	-	ASA 3	BC	P + S	M-S
	10	30	Female	-	ASA 1	AC	P + S	M-S
	11	67	Female	-	ASA 2	TS	P + S + W	M-S
	12	47	Male	C	ASA 1	BC	P + S	M-S
	13	50	Male	-	ASA 2	BC	P + S	M-S
	14	28	Male	C	ASA 1	TS	P + S	M-S + LW
	15	21	Female	-	ASA 1	BC	P + S	M-S
	16	48	Male	-	ASA 2	TS	P + S	M-S
	17	46	Female	-	ASA 1	AC	P + W	M-S
	18	53	Female	-	ASA 1	AC	P + S	M-S
	19	31	Male	-	ASA 1	TS	P + S	M-S
	20	41	Male	-	ASA 1	BC	P + S	M-S
Collected patient data (group A) Controlled cases								
	1	24	Male	-	ASA 1	TS	P + W	II + LW
	2	56	Female	-	ASA 2	BC	P	II + K-L
	3	49	Female	-	ASA 1	TS	P	II
	4	33	Male	-	ASA 2	BC	P	II + K-L
	5	67	Female	-	ASA 2	AC	P + S+ W	II
	6	43	Male	-	ASA 2	BC	P + S + W	II + LW
	7	56	Male	-	ASA 2	BC	P + S	II + K-L
	8	38	Female	-	ASA 1	AC	P + W	II
	9	27	Male	-	ASA 1	TS	W + P + S	II
	10	42	Female	-	ASA 2	TS	P + S	II + LW
	11	54	Female	-	ASA 2	BC	P + S + W	II
	12	53	Male	-	ASA 2	AC	P + W	II
	13	29	Male	-	ASA 1	BC	P	II + LW
	14	62	Male	-	ASA 3	AC	P	II
	15	45	Male	-	ASA 1	TS	P + S	II + LW
	16	39	Male	-	ASA 1	BC	P + S	II + K-L
	17	35	Male	-	ASA 1	BC	P + W	II
	18	66	Male	-	ASA 2	TS	P + S	II + LW

*Group A indicates 18 patients operated by ilioinguinal approach (Il)

*Group B indicates 20 patients operated via modified Stoppa approach (MS)

*P* plate, *S* screw, *LW* lateral window, *K-L* Kocher-Langenbeck

**Table 3:** Operative variables among the two groups

	Group (A) ilioinguinal	Group (B) Stoppa	*p* value
Operative time (min)	211.14 ± 25.0	116.15 ± 21.6	0.086
Mdn (min–max)	200 (100-300)	130 (75-205)	
Blood loss (cc)	856.5 ± 194.2	335 ± 115.4	0.011*
Blood units transfused			0.062
0	3 (17%)	8 (40%)	
1	9 (50%)	8 (40%)	
2	6 (33%)	4 (20%)	
Fixation devices			
AC	Iliac wing plate (75%)	Pelvic brim plate (100%)	
BC	Associated KL (63%)	Lat. window (44%)	
TS	LW (83%)	Lat. window (17%)	

#### Mode of trauma

**Fig. 2:**
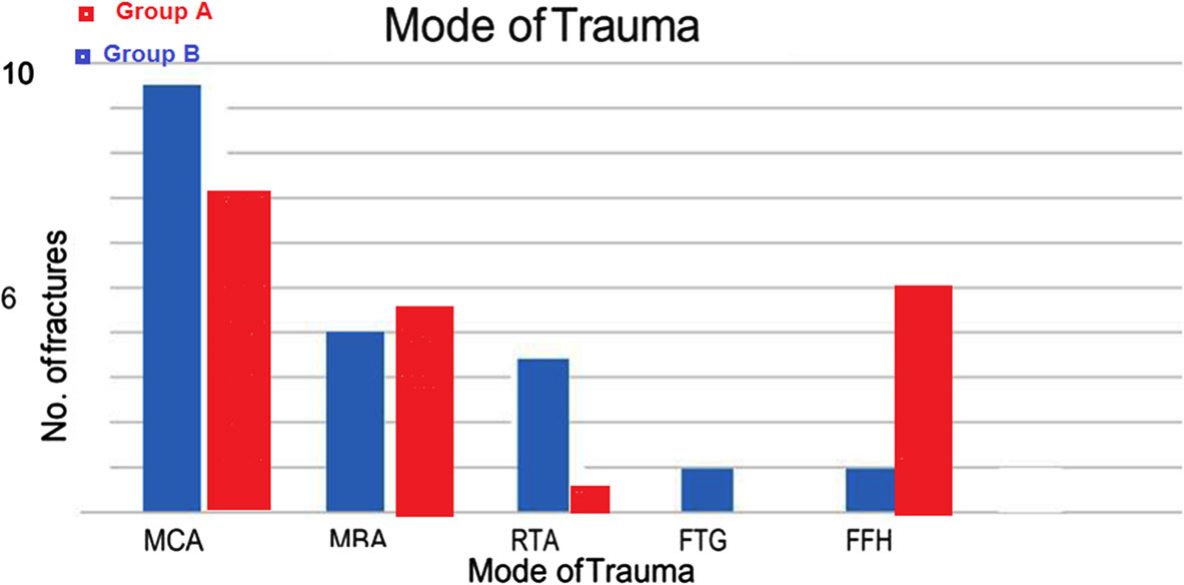
Mode of trauma

In the study groups, 9 patients in group B had motor car accidents (MCA) and 7 in group A, 4 patients had motor bike accidents (MBA) in group B and 5 in group A, 3 had road traffic accidents (RTA) in group B, while 1 only in group A, 2 patients had fall to the ground (FTG), and 2 fell from a height (FFH) in group B in contrast to 6 patients FFH in group A (Fig. 2).

#### Types of acetabular fractures

**Fig. 3:**
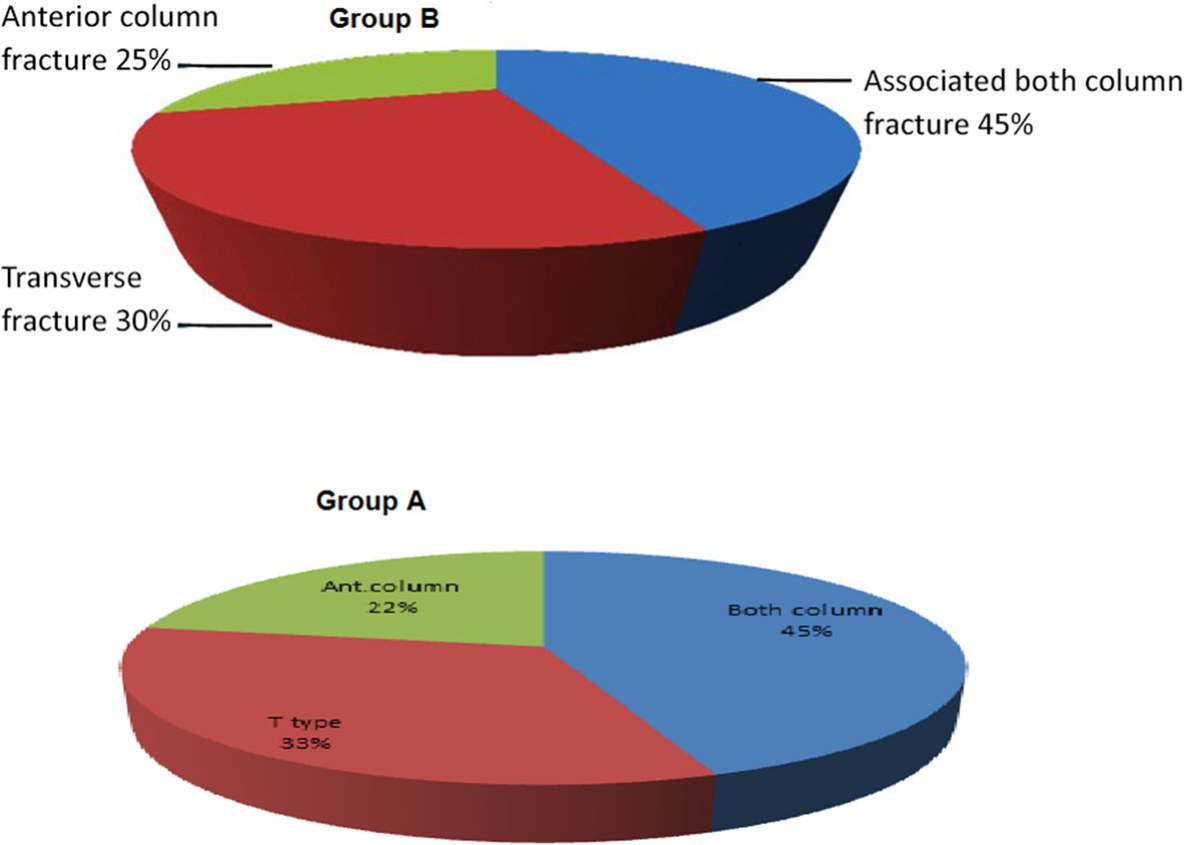
Fracture group classification

Of group B patients, 9 patients (45%) had associated both column fractures, 6 patients (30%) had T type fractures, and 5 patients (25%) had anterior column fractures while of group A patients, 8 patients (45%) had associated both column fractures, 4patients (22%) had T type fractures, and 6 patients (33%) had anterior column fractures as illustrated in Fig. 3.

#### Statistical analysis

Results are expressed as mean ± standard deviation or number (%). Comparison between values of different parameters in the studied groups was performed using Kruskal Wallis test followed by Mann-Whitney test as a post hoc test if significant results are recorded. Comparison between categorical data was performed using chi-square test. SPSS (Statistical Package for the Social Science; SPSS Inc., Chicago, IL, USA) (version 16 windows) was used for data analysis. *P* value less than or equal to 0.05 was considered significant and less than 0.01 was considered highly significant.

### Operative data analysis

Operative variables among the two groups are illustrated in Table 3 which concludes that shorter operative time (duration of surgery), less blood loss, and blood transfusion needed were significantly noticed in group B patients treated with modified Stoppa approach. Regarding fixation devices, Kocher-Langenbeck approach was used associated with ilioinguinal approach in 63% of both column fractures, while lateral window is needed with anterior Stoppa approach in 44% of the same type of fractures. In order to access posterior column, the classic descriptive approach was used alone in 56% of cases with all types of fractures and exclusive with cases of anterior fracture type (Table 2).

### Postoperative mobilization protocol for both groups

As soon as drain removed, in-bed passive hip movement exercises started to begin. Patients were then encouraged to engage in touch-down weight-bearing mobilization as early as possible for the operative side by taking into account each patient’s general condition and concomitant injuries. Until the 8th week postoperatively, touch-down weight bearing was permitted and continued with partial weight bearing with two crutches and then one crutch until full weight bearing was achieved at the 12–16th week by considering each patient’s radiological findings. Patients with severe comminution of the acetabulum were subjected to skeletal traction for 2–3 weeks. Those patients who had bilateral acetabular fractures were advised to get engaged with in-bed and bedside exercises, passive hip movement exercises, and hip strengthening exercises with no weight-bearing until week 6–8 postoperatively. Mobilization was then continued with partial weight-bearing after considering each patient’s radiological findings as follows:

- Static quadriceps exercises and ankle dorsiflexion exercises were started within 24 h after the surgery.
- Passive and active knee exercises while in recumbent position commenced from day 2 postoperative.
- Once the pain had subsided, the patient started gait training on a walker or axillary crutches. Without weight bearing on the affected side.
- Active flexion, extension, and abduction exercises while standing were encouraged. Physical therapy was directed towards regaining muscle strength around the hip and range of motion.
- Limitation of weight bearing was continued for 8-12 weeks postoperatively.
- 12 weeks: Full weight bearing ambulation was permitted only after fracture healing, evident by clinical and radiological union. This was usually achieved by about 12 weeks. Patient was then advised to gradually discard walking aids as tolerated and assessed in postoperative visits (Table 4).

**Table 4:** Timing of follow up visits

Patient discharge	14 days	6 weeks	12 weeks	18 weeks	Every 3-6 months
24 h after drain removal and after ensuring commencement of R.O.M. exercises	Suture removal hip exercises while standing	•Follow up X-rays •Follow up range of motion	•Follow up X-rays •Follow up range of motion •Start weight bearing	Functional assessment	Functional assessment •Commence sports and high demand activities

### Postoperative outcome results

**Fig. 4:**
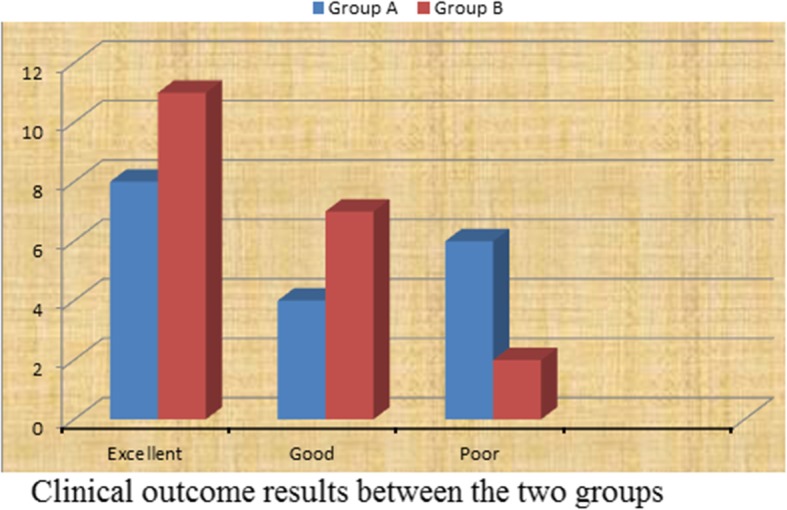
Immediate postoperative radiological outcome

**Fig. 5:**
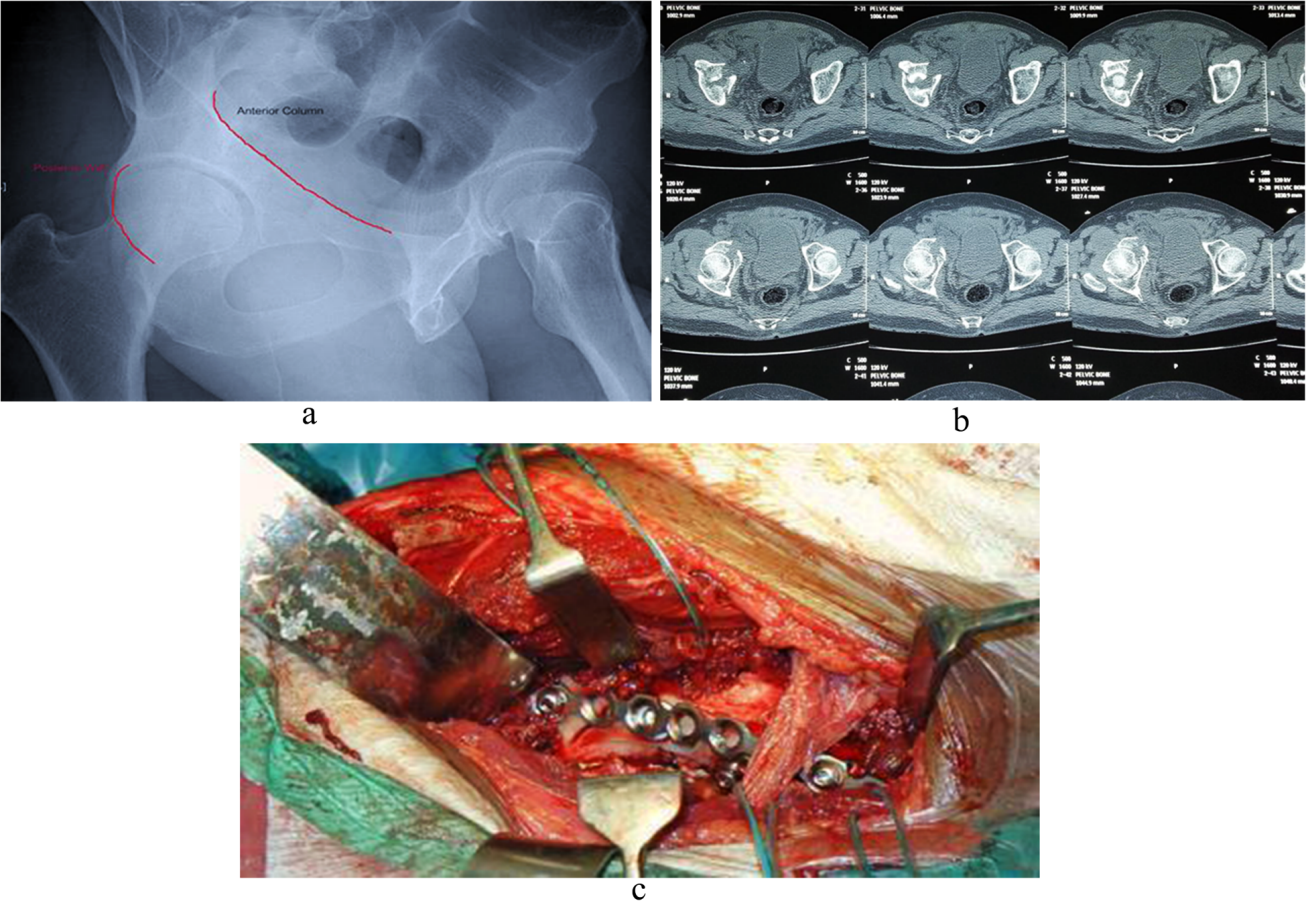
Anterior column fracture AP view (**a**) and axial CT preoperative (**b**) and intraoperative (**c**)

**Fig. 6:**
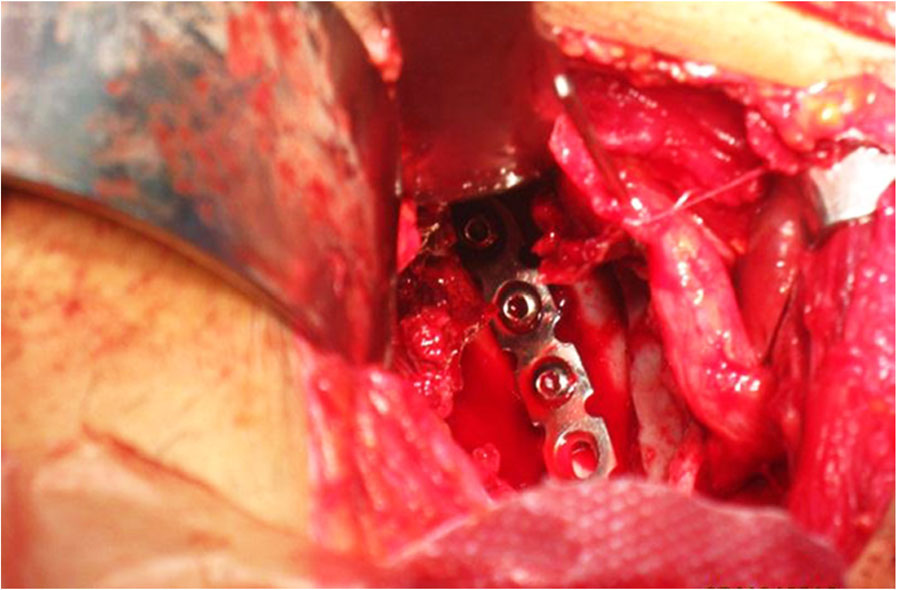
Intra-operative reduction and fixation of anterior column

**Fig. 7:**
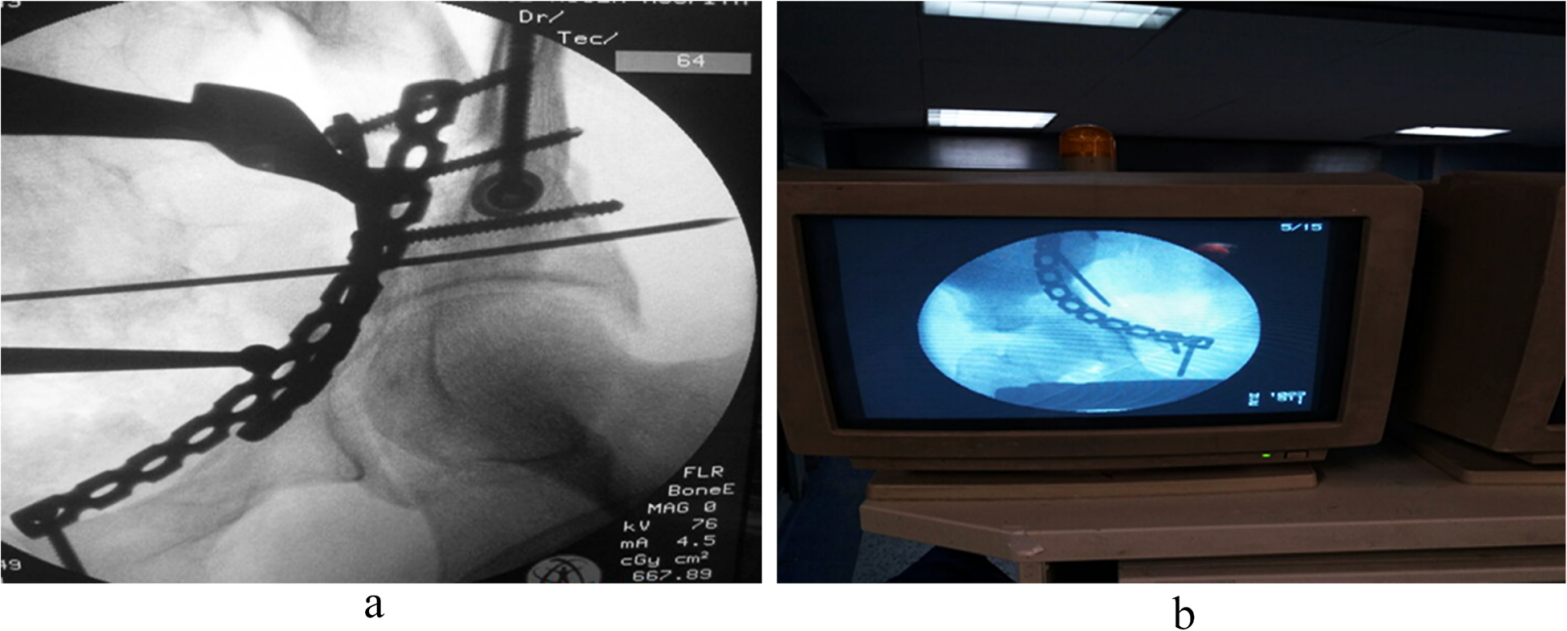
Intraoperative C-arm view; right side Stoppa (**a**) and left side Stoppa (**b**)

Quality of reduction was estimated on anteroposterior (AP) pelvis and Judet views that taken immediately after surgery evaluated by Matta criteria; comparisons between the two groups are illustrated in Table 5 noticed that the quality of reduction is excellent with 75% of patients in group B treated with modified Stoppa approach (*p* = 0.03). Excellent clinical outcome was observed in 8 patients of group A, versus 11 patients of group B. Good in 4 patients of group A, versus 7 patients of group B, and poor clinical outcome in 6 patients of group A, versus 2 patients of group B (*p* = 0.05) (Fig. 4). Incidence of late postoperative residual subluxation of the femoral head was 55% in patients of group A, which was significantly low (30%) in patients of group B those treated with modified Stoppa (Table 6). The mean modified Merle D’Aubinge and Postel score in both groups according to fracture types showed an excellent clinical results in patients of modified Stoppa approach especially with anterior column fracture type compared to patients underwent ilioinguinal approach for the same fracture type (Table 7). Lastly, postoperative complications were compared in the two groups emphasized that modified Stoppa approach is associated with significantly less incidence of complications that made it; the approach of choice when surgery is required (Table 8). Preoperative and postoperative radiology are illustrated in Figs. 5, 6 and 7.

**Table 5:** Clinical results related according to the quality of reduction (*p* = 0.03)

Result (Matta criteria)	A*	%	B*	%
**Anatomical (0-1 mm displacement) didisplacement**	**9**	**50**	**15**	**75**
**Imperfect (2-3 mm displacement)**	6	33	3	15
**Poor (< 3 mm displacement)**	3	17	2	10
**Total**	18	100	20	100

*Group A indicates 18 patients operated by ilioinguinal approach

*Group B indicates 20 patients operated via modified Stoppa approach

**Table 6:** Incidence of late postoperative residual subluxation of the femoral head

Groups	Frequency	Percentage	*p* value
Group (A) (*n* = 18) ilioinguinal	10	55	0.002^**^
Group (B) (*n* = 20) Stoppa	6	30

**Table 7:** Functional score in both groups according to the modified Merle D’Aubigné and Postel score

Type or fractures	Group (A) ilioinguinal	Group (B) Stoppa
Anterior column	16 (good)	17.7 (excellent)
Transverse	15 (good)	16 (good)
Both column	14 (fair)	15 (good)

**Table 8:** Complications of postoperative acetabular fractures

Complications	Group A (no. of patients)	Group B (no. of patients)	Management
*Intra operative complications*			
Corona mortis injury	2 (11%)	1 (5%)	Packing and ligation in 1 patient
Obturator artery injury	1 (5.5%)	0%	Packing and ligation
External iliac vein injury	1 (5.5%)	0%	Primary repair
Superior gluteal artery injury	1 (5.5%)	1 (5%)	Packing and embolisation
*Postoperative complications*			
Obturator nerve injury	1 (5.5%)	0%	Recovered in 3-6 months in 20 patients
Femoral Nerve palsy	1 (5.5%)	0%	Partial recovery in 1 patient
Deep infection	2 (11%)	1 (5%)	Debridement and antibiotics in 2 patients
Foot drop	2(11%)	1 (5.5%)	Recovered in 6-12 months
Superficial infection	4 (22%)	2 (10%)	Dressing and antibiotics
Deep vein thrombosis	3 (33%)	1 (5%)	Chemical prophylaxis in 1 patients
Intra articular screw	3 (33%)	2(10%)	Removed
Sciatic nerve palsy	1 (5.5%)	0%	Recovered in 6 weeks
Seroma at operative site	1 (5.5%)	1 (5%)	Treated operatively, no infection was found
Peritoneum breach	1 (5.5%)	0%	Wound was closed without sequelae
Wound dehiscence	2(11%)	1 (5%)	Surgical closure done
Delayed wound healing	2(11%)	1 (5%)	Healed in 3 weeks with infrared heat lamp treatment
*Late complications*			
Hip joint arthritis	6 (66%)	5 (25%)	
Ectopic bone formation	4 (22%)	3 (15%)	Conservative in 2 patients with full range of motion
Avascular necrosis femur head	1 (5.5%)	0%	
Loss of reduction	2 (11%)	1 (5%)	THR done in 2 patients
Rectus atrophy without hernia	1 (5.5%)	1 (5%)	
Lateral inguinal hernia	2 (11%)	1 (5%)	Repair done

## Discussion

Acetabular fractures are difficult to treat due to the difficulties associated with the surgical approach, associated organ injuries, and complex nature of the fracture itself. This study evaluated the clinical outcomes of the modified Stoppa approach for reduction in acetabular fractures.

In the patient group of our study, the number of male patients was higher than female patients. Similar to our study, 16 studies on 609 patients to evaluate the Stoppa approach revealed that the male-female ratio was available for 566 patients, which was same as our study [5, 11–23]. Data for the remaining patients were unavailable due to various reasons. But the common fact in all these studies was the number of male patients was higher than that of female patients.

The range of age in the 11 studies we cited was 10-88 years [5, 11–15, 17, 21–24]. The average age was 49.3 years for Elmadag et al., 41.88 years for Shazar et al., and 55.88 years for Dailey et al. [11, 16–20]. The mean age in our study was much lower (36.8 ± 8.42 (range 20-73) years) compared to these observations. The difference could be mainly because our study had a smaller age range.

The most common mode of injury was RTA (129 patients), which was followed by FFH (108 patients), industrial accident (6 patients), crush injury (3 patients), and sport injury (1 patient) as observed in 6 studies (271patients) [12–15, 17, 21, 23]. Also, Cole et al. [3] reported RTA as the commonest mode of injury (85%). In contrast, the most common mode of injury in our study was MCA (9 patients).

### Classification of fractures

When 456 patients from 11 studies were analyzed using Judet and Letournel classification, the most common fractures were associated with both column (128), anterior column with posterior hemi transverse (89), anterior column (84), T type (54), transverse (53), transverse with posterior wall (29), and anterior column (13). Similar to our study, the majority of fractures were associated both column fractures (45%).

### Delay in surgery

Time delay for surgery ranged from 0 day to 30 days in 8 studies [5, 11–23]. In comparison, Dailey et al. reported a mean time of 4.83 ± 3.74 from injury to surgery [20]. There were many reasons affecting the preoperative time in our study. The most important was the time between the actual trauma and the date at which the patient presented to our hospital. Five patients included in this study presented to us after 4-7 days of trauma. Another factor was the availability of blood units for intraoperative transfusion. Three patients were of blood group B– and two patients were of blood group AB–; this caused significant delay in the operative date. Another factor worth mentioning is the waiting list for the operative theater.

### Surgical time

In the 7 studies we cited, the surgical time ranged from 80 min to 568 min [5, 11–15, 17, 21–24]. Anderson et al. reported the surgical time from 3 h to 8 h 48 min, including the time until the physician reviews the after-surgery radiographs [5]. The mean operative time for our study in group A was 211.14 ± 25.0 min, which was longer compared to group B was 116.15 ± 21.6 min.

Estimated blood loss reported in the 9 studies ranged from 100-5000 ml [5, 11–15, 17, 21–24]. Our results were also in agreement with these observations. In addition, the blood loss (856.5 ± 194.2) and need for intraoperative transfusion (100%) in group A, while less in group B (335 ± 115.4) (Table 3).

### Outcome

Twelve studies (408 patients) evaluated clinical outcomes in terms of postoperative fracture reduction using Matta’s method [5, 11–15, 17, 21–24]. They observed anatomic reduction (≤ 1 mm) in 290 patients, imperfect reduction (> 1 to < 3 mm) in 77 patients and poor reduction (≥ 3 mm) in 34 patients while anatomic or imperfect reduction in 7 patients.

Sagi et al. observed that the patients associated with both column fractures show poor fracture reduction (75%) [6]. Patients with anterior column fracture achieved the highest percentage of excellent reduction (92%), whereas patients with transverse fractures had the lowest percentage (67%). Results of Shazar et al. were also similar; except for the patients with anterior column with posterior hemitransverse fractures achieved the highest reduction (92.9%) of anatomical reduction was achieved in anterior column with posterior hemitransverse fractures [16].

#### Harris hip score

Harris hip score was evaluated by four studies [10, 13, 15, 18]. In three studies (82 patients), Harris hip score was 35 excellent, 34 good, eight fair, and five poor [10, 13, 18]. Laflamme et al. found mean Harris hip score 81 (range 51–100) in a series of 9 patients [15].

#### Merle D’Aubigné score

In four studies (133 patients) Merle D’Aubigné score was 58 excellent, 59 good, eight fair, eight poor [10, 13, 15, 18, 24, 25]. Isaacson et al. in their study described 12 very good, two good, four medium, one fair, and three poor results according to Merle D’Aubigné score [19, 24].

### Complications

The most frequent early complication from initial trauma reported is obturator nerve injury and the late complication from initial trauma is hip joint arthritis. Soni et al. reported an improvement in certain acetabular fractures and also concluded that the Stoppa approach may have lower rate of complications compared to extrapelvic extensile approaches. In comparison, Kima et al. estimated that the modified Stoppa approach is associated with 9% rate of obturator nerve injury from initial trauma [24]. The modified Stoppa approach may be helpful in releasing obturator nerve and consequently in obturator nerve injury (Table 8).

Therefore, the modified Stoppa approach is strongly recommended in patients with two-column acetabular fractures in the case of preoperative detection of obturator nerve injury.

### Access to quadrilateral plate

In this study, we used the modified Stoppa approach along with lateral window. Soni et al. recommended using modified Stoppa approach as a substitute to total hip arthroplasty to support the quadrilateral plate in patients with fracture acetabulum involving anterior column and quadrilateral surface.

### Access to posterior column

Modified Stoppa approach with lateral window of ilioinguinal enhances reduction and fixation of posterior column and quadrilateral surface with a better access. This approach is also helpful in the fixation of anterior column fracture extending into posterior column 8, 9, 12.

Isaacson et al. reported a good functional outcome in 36 patients with acetabular fractures extending to posterior column after treating them with this approach; they also reported a lower rate of complications [6]. Additionally, the anterior approach alone has been reported to be difficult for the treatment but is a feasible option.

### Variations of the approach

The modified Stoppa approach is either combined with other approaches or modified in the practice [9, 13, 15]. Rocca et al. combined it with proximal and lateral window of ilioinguinal approach (anterior combined endopelvic; ACE) [8]. The Stoppa approach was modified by Sagi et al. as anterior intrapelvic (AIP) approach. The Stoppa approach has resulted in a reduction in fractures along with comparable complication rates [6].

### Comparison with ilioinguinal approach

Outcomes of Stoppa approach have been compared in five studies with ilioinguinal approach with respect to fixing the fracture acetabulum [13, 17–19, 21]. Rocca et al. compared ACE approach (34 patients) with ilioinguinal approach (42 patients) to treat acetabular fracture [9]. They reported better clinical outcomes with ACE approach compared to the ilioinguinal approach. They also reported a lower complication rate with the ACE approach. In contrast, Hammad AS et al. reported a similar reduction in fracture and similar clinical scores with ACE and ilioinguinal approach [19]. Stoppa approach provides direct buttressing of quadrilateral surface and associated both column fractures. But associating the Stoppa approach with middle window of ilioinguinal approach improves access to anterior wall and transversely oriented fractures.

In the contrast, Elmadag et al. reported that the Stoppa approach does not provide any improvement in associated complication rates and bleeding compared to the ilioinguinal approach but has a cosmetically better scar [11]. Ma et al. also reported similar results but better operative time, wound drainage, and lesser blood loss, suggesting lesser blood transfusion with Stoppa approach [17].

Stoppa approach improves visualization in lateral compression injuries and allows treatment of bilateral fractures with single incision. Additionally, Shazar et al. reported a higher rate of anatomical reduction in lesser surgical time and comparable complication rate with Stoppa approach compared to ilioinguinal approach [16]. Soni et al. concluded that modified Stoppa approach is a better option for treating fracture acetabulum [23]. A lateral window without exposing the neurovascular bundle to treat the fractures extending to the iliac crest is another advantage of the Stoppa approach.

Finally, this study has limitations such as having limited number of patients over long study period, and short term follow-up.

## Conclusion

The study reports favorable results by the modified Stoppa approach in the treatment of the anterior acetabular fractures as it improves visualization in lateral compression injuries and allows treatment of both column fractures with single incision. Thus, this approach can be a viable option for the classic ilioinguinal approach that was long used in developing countries. Further research work is recommended on a larger scale through comparative studies between the two surgical modalities for different acetabular fracture types.

## Data Availability

Available data and materials presented in the main manuscript and additional supporting files in soft-readable format available only upon reasonable request and after consent for publication.

## Abbreviations

ACE: Anterior combined endopelvic approach
AIP: Anterior intrapelvic approach
DVT: Deep venous thrombosis
FFH: Falling from high
FTG: Falling to the ground
MBA: Motor bike accidents
MCA: Motor car accidents
RTA: Road traffic accidents
